# Dynamic molecular signatures of acute myocardial infarction based on transcriptomics and metabolomics

**DOI:** 10.1038/s41598-024-60945-3

**Published:** 2024-05-03

**Authors:** Xuejiao Wang, Guang Yang, Jun Li, Chao Meng, Zengming Xue

**Affiliations:** 1 Department of Cardiology, Guang’anmen Hospital, China Academy of Chinese Medical Sciences, No. 5 Beixiange, Xicheng District, Beijing, 100053 China; 2 Department of Cardiology, Langfang People’s Hospital, Hebei Medical University, No. 37, Xinhua Road, Langfang, 065000 China

**Keywords:** Cardiovascular diseases, Acute coronary syndromes, Transcriptomics

## Abstract

Acute myocardial infarction (AMI) commonly precedes ventricular remodeling, heart failure. Few dynamic molecular signatures have gained widespread acceptance in mainstream clinical testing despite the discovery of many potential candidates. These unmet needs with respect to biomarker and drug discovery of AMI necessitate a prioritization. We enrolled patients with AMI aged between 30 and 70. RNA-seq analysis was performed on the peripheral blood mononuclear cells collected from the patients at three time points: 1 day, 7 days, and 3 months after AMI. PLC/LC–MS analysis was conducted on the peripheral blood plasma collected from these patients at the same three time points. Differential genes and metabolites between groups were screened by bio-informatics methods to understand the dynamic changes of AMI in different periods. We obtained 15 transcriptional and 95 metabolite expression profiles at three time points after AMI through high-throughput sequencing. AMI-1d: enrichment analysis revealed the biological features of 1 day after AMI primarily included acute inflammatory response, elevated glycerophospholipid metabolism, and decreased protein synthesis capacity. Phosphatidylcholine (PC) and phosphatidylethanolamine (PE) might stand promising biomarkers to differentiate post-AMI stage. Anti-inflammatory therapy during the acute phase is an important direction for preventing related pathology. AMI-7d: the biological features of this stage primarily involved the initiation of cardiac fibrosis response and activation of platelet adhesion pathways. Accompanied by upregulated TGF-beta signaling pathway and ECM receptor interaction, GP5 help assess platelet activation, a potential therapeutic target to improve haemostasis. AMI-3m: the biological features of 3 months after AMI primarily showed a vascular regeneration response with VEGF signaling pathway, NOS3 and SHC2 widely activated, which holds promise for providing new therapeutic approaches for AMI. Our analysis highlights transcriptional and metabolomics signatures at different time points after MI, which deepens our understanding of the dynamic biological responses and associated molecular mechanisms that occur during cardiac repair.

## Introduction

With the process of population aging, cardiovascular diseases have become the leading non-communicable chronic cause of death^1^. According to the report of Global Burden of Disease Study 2019, there were 18.5 million deaths globally due to cardiovascular diseases, accounting for about one-third of all global deaths^2^. Among the 2.45 million deaths caused by cardiovascular diseases in China, approximately 1 million people (40.5%) died from myocardial infarction. Acute myocardial infarction (AMI) is the most common acute and critical cardiovascular disease in clinical practice, with high incidence, mortality, and poor prognosis^3^. Percutaneous coronary intervention (PCI) and fibrinolysis are key treatment methods for AMI, while medications, including anti-platelet drugs, receptor blockers, ACE inhibitors, calcium channel blockers, and statins, are recommended for clinical practice and can significantly reduce the mortality and incidence associated with myocardial infarction^4,5^. As a long-term pathophysiological stage, post-AMI inevitably occurs gradual recovery, leading to certain scar tissue or other structural changes^6^. With the mortality rate of myocardial infarction patients has significantly declined^7^, there is an increasing number of patients experiencing post AMI ventricular remodeling and heart failure as the long-term complication. Therefore, understanding the dynamic characteristics of AMI evolution may give a deep insights into the pathogenesis and progression of the disease.

For myocardial infarction, a disease model with complex pathophysiology and multi-factor regulation, it is suitable to carry out high-throughput omics studies to clarify the expression differences of gene, protein and metabolic profiles. After myocardial infarction, remodeling is often described as a process involving three major overlapping stages: inflammation, proliferation, and maturation^8^. There have been already some omic studies on the dynamic evolution of myocardial infarction, basic and clinical. As is reported that at day 1, 3, and 5 after murine myocardial infarction, cardiac Ly6G^+^ neutrophils could be delineated into 6 distinct clusters with specific time-dependent patterning and proportions via single-cell transcriptomics^9^. In addition, via rat metabolomics at 1 h, 1 day and 10 day post myocardial infarction, a time-dependent increase or decrease in polar and lipid metabolite levels was measured. Sadenosylmethionine (SAM) concentration and SAM/S-adenosylhomocysteine (SAH) ratio gradually decreased and were significantly downregulated 10 days after MI, which is related to energy-dependent metabolic pathways^10^. Besides, clinical proteomic analysis have shown certain proteins may serve as biomarkers for the early stages of AMI and monitor early cardiac ischemic recovery^11^. And 9 CpG sites can differentiate MI significantly via DNA methylationin^12^. Metabolomics found the dynamic change of eicosanoic acid content over time may be involved in the cardiac injury and repair after PCI^13^.

The above studies searched for the latest molecular markers and changes through dynamic proteome, metabolome, methylation, but the time point was limited to 72 h or 28 days, ignoring late remodeling, which is of great significance for poor prognosis of myocardial infarction. In this study, we included clinical patients at 1 day, 7 days, and 3 months after MI and used transcriptomics and metabolomics techniques to identify differential genes and metabolites. Additionally, we employed bioinformatics analysis to uncover significant biological processes and signaling pathways that undergo.

## Results

### Quality assessment of raw data

**Figure 1:**
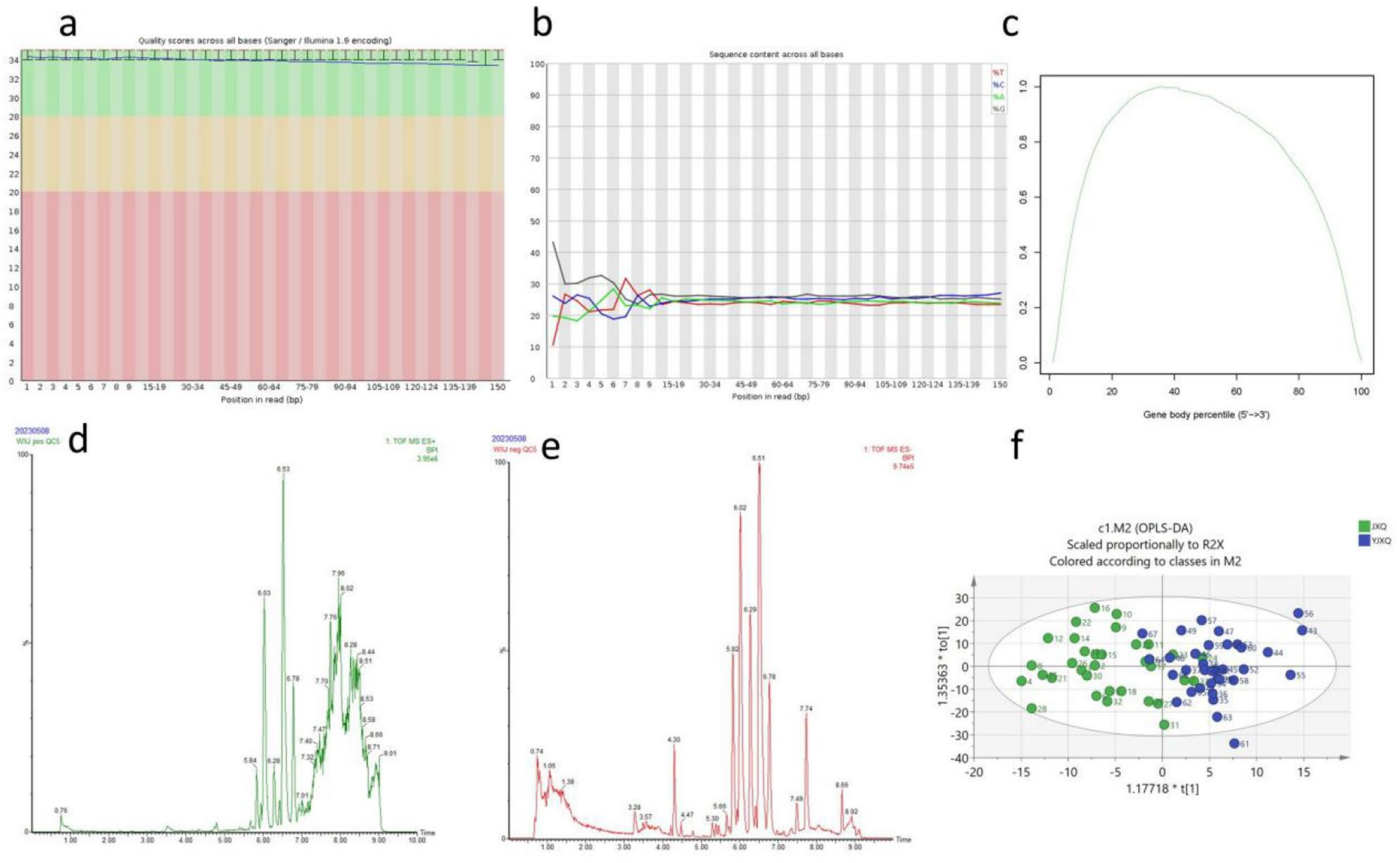
Quality assessment of raw data: (**a**) Quality scores across all bases. (**b**) Sequence content across all bases. (**c**) Map of gene coverage analysis results. (**d**) Typical positive ion chromatogram. (**e**) Typical chromatogram of negative ions. (**f**) OPLS-DA results.

First, we conducted quality assessment of the transcriptomic raw data using the FastQC software. The relevant results are shown in Table 1. Q20 represents a base call error probability of less than 1%. The sequencing data achieved Q20 or above for 98.89% to 99.19% of the bases, indicating a low base call error rate (Fig. 1A). The base composition analysis showed stable levels of A, T, G, and C, indicating similar frequencies of the four bases and a stable sequencing process (Fig. 1B). The gene coverage analysis indicated a symmetrical distribution of base composition, suggesting good randomness in the sequencing data (Fig. 1C). Next, we used the Fastp software to filter the raw data and obtained clean data for subsequent bioinformatics analysis. For metabolomics, the peak ion flow charts for positive and negative ions are shown in Fig. 1D,E, respectively. The OPLS-DA analysis showed clustering of samples within groups and dispersion between groups, indicating good predictive capability of the model (Fig. 1F).

**Table 1:** Quality assessment of raw data.

SampleID	Total_Reads	Total_Bases	N_Reads%	C%	G%	Error%	Q20%	Q30%	GC%
AMI-3m1	35,296,166	5,234,307,935	0.74	25.39	26.14	0.0365	99.06	96.59	51.53
AMI-3m2	28,485,090	4,151,232,838	0.62	23.38	23.72	0.038	98.89	96	47.1
AMI-3m3	32,278,426	4,724,733,065	0.47	22.6	22.91	0.0362	99.18	97.06	45.51
AMI-3m4	21,916,882	3,222,401,303	0.92	23.79	24.18	0.0353	99.15	96.64	47.97
AMI-3m5	39,021,874	5,761,651,232	0.59	24.32	24.74	0.036	99.17	97	49.05
AMI-3m6	37,102,608	5,517,709,003	0.77	23.45	23.85	0.0368	98.98	96.29	47.3
AMI-3m7	34,425,016	5,138,373,994	0.95	24.12	24.52	0.0355	99.07	96.58	48.64
AMI-1d1	39,155,458	5,832,073,361	0.77	24.71	25.32	0.0369	98.98	96.34	50.02
AMI-1d2	32,867,434	4,874,873,624	0.62	23.92	24.44	0.0363	99.11	96.8	48.36
AMI-1d3	34,591,768	5,142,395,322	0.96	23.64	24.08	0.0355	99.08	96.58	47.72
AMI-7d1	40,257,524	5,948,581,774	0.81	24.16	24.59	0.0349	99.21	97.06	48.74
AMI-7d2	47,644,776	7,086,277,473	0.03	23.36	23.73	0.041	98.87	95.86	47.09
AMI-7d3	53,850,526	7,997,086,104	0.03	23.84	24.17	0.0402	99.01	96.34	48.01
AMI-7d4	66,601,968	9,819,305,841	0.04	23.86	24.23	0.0383	99.19	97.12	48.09
AMI-7d5	38,971,158	5,793,028,259	0.92	24.19	24.73	0.0354	99.08	96.65	48.92

### Transcriptional and metabolomic results between AMI-1d and AMI-7d

**Figure 2:**
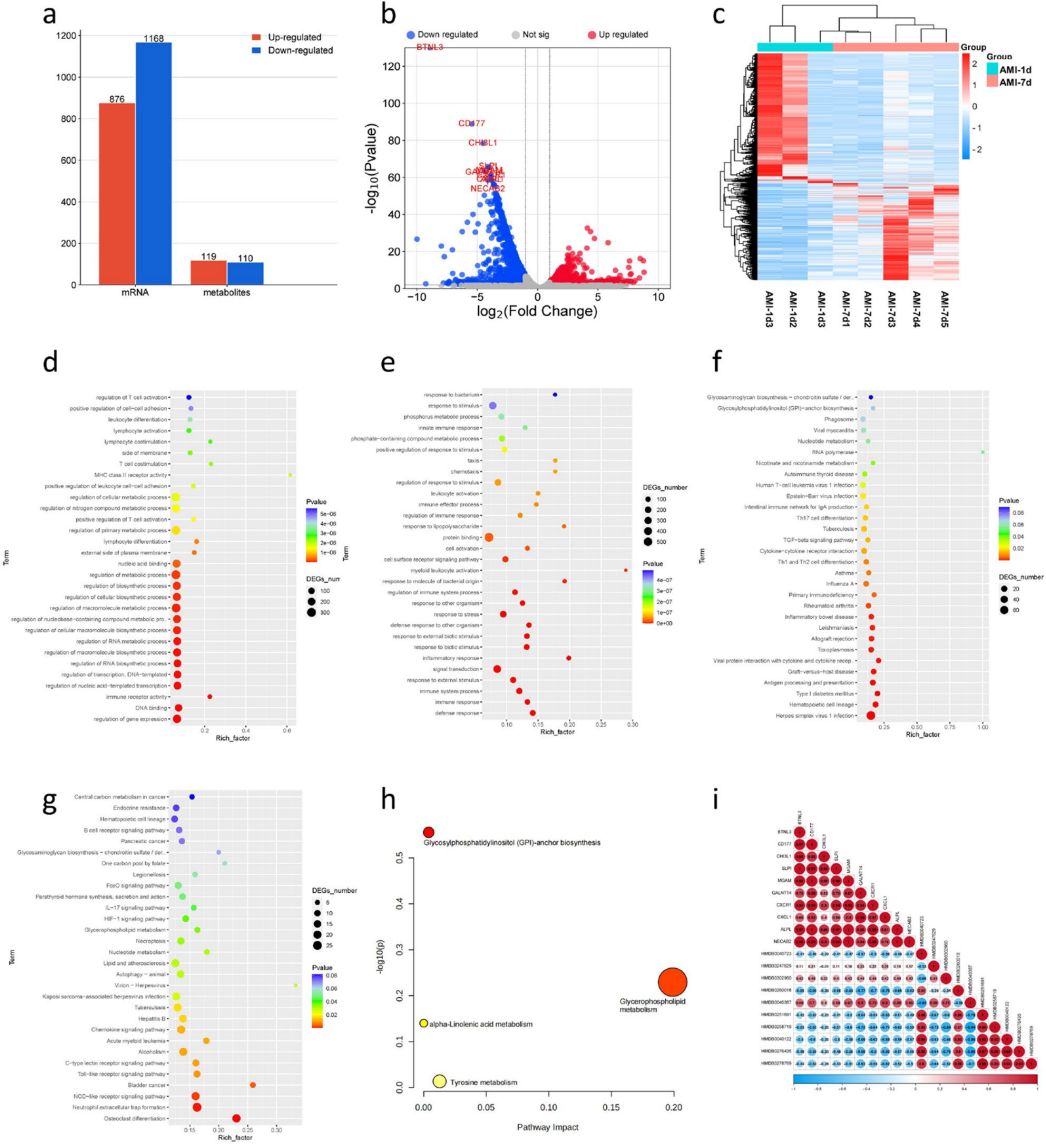
Transcriptional and metabolomic results between AMI-1d and AMI-7d: (**a**) Histogram of DEGs between AMI-1d and AMI-7d; (**b**) Volcano plot of DEGs between AMI-1d and AMI-7d; (**c**) Clustering heat map of DEGs between AMI-1d and AMI-7d; (**d**) Go enrichment analysis of up-regulated DEGs; (**e**) Go enrichment analysis of down-regulated DEGs; (**f**) KEGG enrichment analysis of up-regulated DEGs; (**g**) KEGG enrichment analysis of down-regulated DEGs; (**h**) KEGG enrichment analysis of DMs; (**i**) Correlation analysis between the top 10 DEGs and DMs.

Compared to AMI-1d, there were 876 upregulated genes and 1168 downregulated genes under the condition of FC > 2 and FDR < 0.05 (Fig. 2A). The top 10 genes ranked by P-value were: BTNL3, CD177, CHI3L1, SLPI, MGAM, GALNT14, CXCR1, CXCL1, ALPL, NECAB2 (Fig. 2B). The list of related genes can be found in Table S1. The heatmap analysis revealed distinct differences in gene expression trends between the two groups (Fig. 2C). To better understand the differences in the biological functions of genes between AMI-1d and AMI-7d, we conducted functional and pathway enrichment analyses separately for upregulated and downregulated DEGs. The results of GO enrichment showed that upregulated DEGs in AMI-7d were enriched in biological processes such as regulation of RNA biosynthetic process, regulation of nucleic acid-templated transcription, regulation of macromolecule biosynthetic process, regulation of RNA metabolic process, regulation of gene expression, regulation of transcription, DNA-templated, and regulation of macromolecule metabolic process (Fig. 2D). Downregulated DEGs in AMI-7d were enriched in biological processes such as defense response, immune response, immune system process, response to external stimulus, signal transduction, and inflammatory response (Fig. 2E). The KEGG enrichment results showed that upregulated DEGs in AMI-7d were enriched in pathways such as Herpes simplex virus 1 infection, Hematopoietic cell lineage, Type I diabetes mellitus, Antigen processing and presentation, Viral protein interaction with cytokine and cytokine receptor, Graft-versus-host disease, Toxoplasmosis, Inflammatory bowel disease, Leishmaniasis, and Allograft rejection (Fig. 2F). Downregulated DEGs in AMI-7d were enriched in pathways such as NOD-like receptor signaling pathway, Chemokine signaling pathway, C-type lectin receptor signaling pathway, Toll-like receptor signaling pathway, Nucleotide metabolism, Lipid and atherosclerosis, HIF-1 signaling pathway, Glycerophospholipid metabolism, Autophagy, IL-17 signaling pathway, and FoxO signaling pathway (Fig. 2G).

Next, we used UPLC-Q-TOF-MS technology to detect the differences in metabolites between AMI-1d and AMI-7d. We performed multivariate statistical analysis using the OPLS-DA model and calculated P-values using analysis of variance. Metabolites with P < 0.05 and VIP > 1 were selected as DMs. A total of 229 DMs were identified between AMI-1d and AMI-7d, including 113 in negative ion mode and 116 in positive ion mode. Among them, 119 were upregulated and 110 were downregulated in AMI-7d. The list of related metabolites can be found in Table S2. The top 10 metabolites ranked by P-value were HMDB0302960, HMDB0260018, HMDB0040723, HMDB0040122, HMDB0276435, HMDB0040387, HMDB0251691, HMDB0258719, HMDB0247629, and HMDB0278769. We imported these 229 DMs into the MetaboAnalyst database for pathway analysis, and the enriched pathways included Glycerophospholipid metabolism and Glycosylphosphatidylinositol (GPl)-anchor biosynthesis (Fig. 2H). To analyze the relationship between the DEGs and DMs, we performed correlation analysis between the top 10 DEGs and DMs selected based on their P-values. The results of Pearson correlation analysis showed that there were two metabolite-gene pairs, HMDB0040387 (Acrimarine I)-CXCL1 and Acrimarine I-GALNT14, with correlation coefficients ≥ 0.8 (Fig. 2I).

### Transcriptional and metabolomic results between AMI-3m and AMI-7d

**Figure 3:**
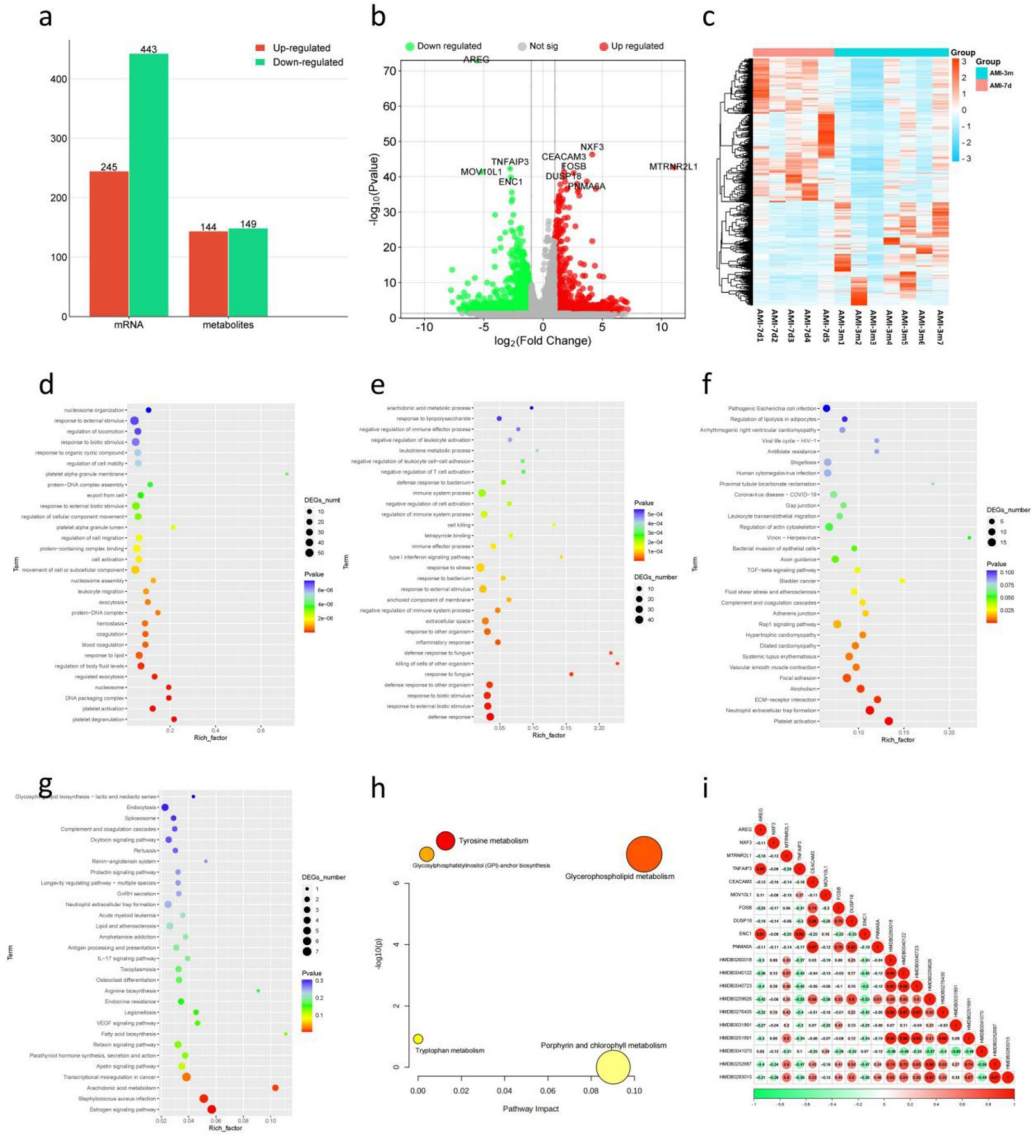
Transcriptional and metabolomic results between AMI-7d and AMI-3m: (**a**) Histogram of DEGs between AMI-7d and AMI-3m; (**b**) Volcano plot of DEGs between AMI-7d and AMI-3m; (**c**) Clustering heat map of DEGs between AMI-7d and AMI-3m; (**d**) Go enrichment analysis of up-regulated DEGs; (**e**) Go enrichment analysis of down-regulated DEGs; (**f**) KEGG enrichment analysis of up-regulated DEGs; (**g**) KEGG enrichment analysis of down-regulated DEGs; (**h**) KEGG enrichment analysis of DMs; (**i**) Correlation analysis between the top 10 DEGs and DMs.

Compared to AMI-7d, there were 245 upregulated genes and 443 downregulated genes in AMI-3m (Fig. 3A). The top 10 genes ranked by P-value were AREG, NXF3, MTRNR2L1, TNFAIP3, CEACAM3, MOV10L1, FOSB, DUSP18, ENC1, and PNMA6A (Fig. 3B). The list of related genes can be found in Table S3. The heatmap showed distinct expression patterns between the two groups of genes (Fig. 3C). To better understand the differences in the biological functions and pathways of genes between AMI-3m and AMI-7d, we conducted enrichment analysis for upregulated and downregulated DEGs separately. The GO enrichment analysis revealed that downregulated genes in AMI-3m were enriched in biological processes such as platelet degranulation, platelet activation, regulated exocytosis, regulation of body fluid levels, coagulation, blood coagulation, response to lipid, exocytosis, hemostasis, and leukocyte migration (Fig. 3D). Upregulated genes in AMI-3m were enriched in biological processes such as defense response, response to external biotic stimulus, response to biotic stimulus, defense response to other organism, killing of cells of other organism, defense response to fungus, response to fungus, inflammatory response, response to other organism, and negative regulation of immune system process (Fig. 3E). The KEGG enrichment analysis revealed that downregulated genes in AMI-3m were enriched in pathways such as platelet activation, dilated cardiomyopathy, hypertrophic cardiomyopathy, vascular smooth muscle contraction, Rap1 signaling pathway, complement and coagulation cascades, and fluid shear stress and atherosclerosis (Fig. 3F). Upregulated genes in AMI-3m were enriched in pathways such as estrogen signaling pathway, arachidonic acid metabolism, retrograde endocannabinoid signaling, biosynthesis of secondary metabolites, PI3K-Akt signaling pathway, TGF-beta signaling pathway, calcium signaling pathway, and VEGF signaling pathway (Fig. 3G).

In terms of the metabolome, a total of 293 DMs (P.value < 0.05, VIP > 1) were identified between AMI-7d and AMI-3m. Among them, 144 metabolites were upregulated in AMI-3m, while 149 metabolites were downregulated (Fig. 3A). The list of related metabolites can be found in Table S4. The top 10 DMs ranked by P-value were HMDB0260018, HMDB0040122, HMDB0040723, HMDB0276435, HMDB0259626, HMDB0031891, HMDB0251691, HMDB0041070, HMDB0252687, and HMDB0283015.We imported all the DMs into the MetaboAnalyst database for pathway analysis and obtained a total of four enriched metabolic pathways. These pathways include glycerophospholipid metabolism, porphyrin and chlorophyll metabolism, glycosylphosphatidylinositol (GPI)-anchor biosynthesis, and tryptophan metabolism (Fig. 3H). We selected the top 10 genes and metabolites based on their P-values and performed correlation analysis. The results of Pearson correlation analysis showed that there was a correlation coefficient of ≥ 0.8 between HMDB0259626 and DUSP18 (Fig. 3I).

### Transcriptional and metabolomic results between AMI-3m and AMI-1d

**Figure 4:**
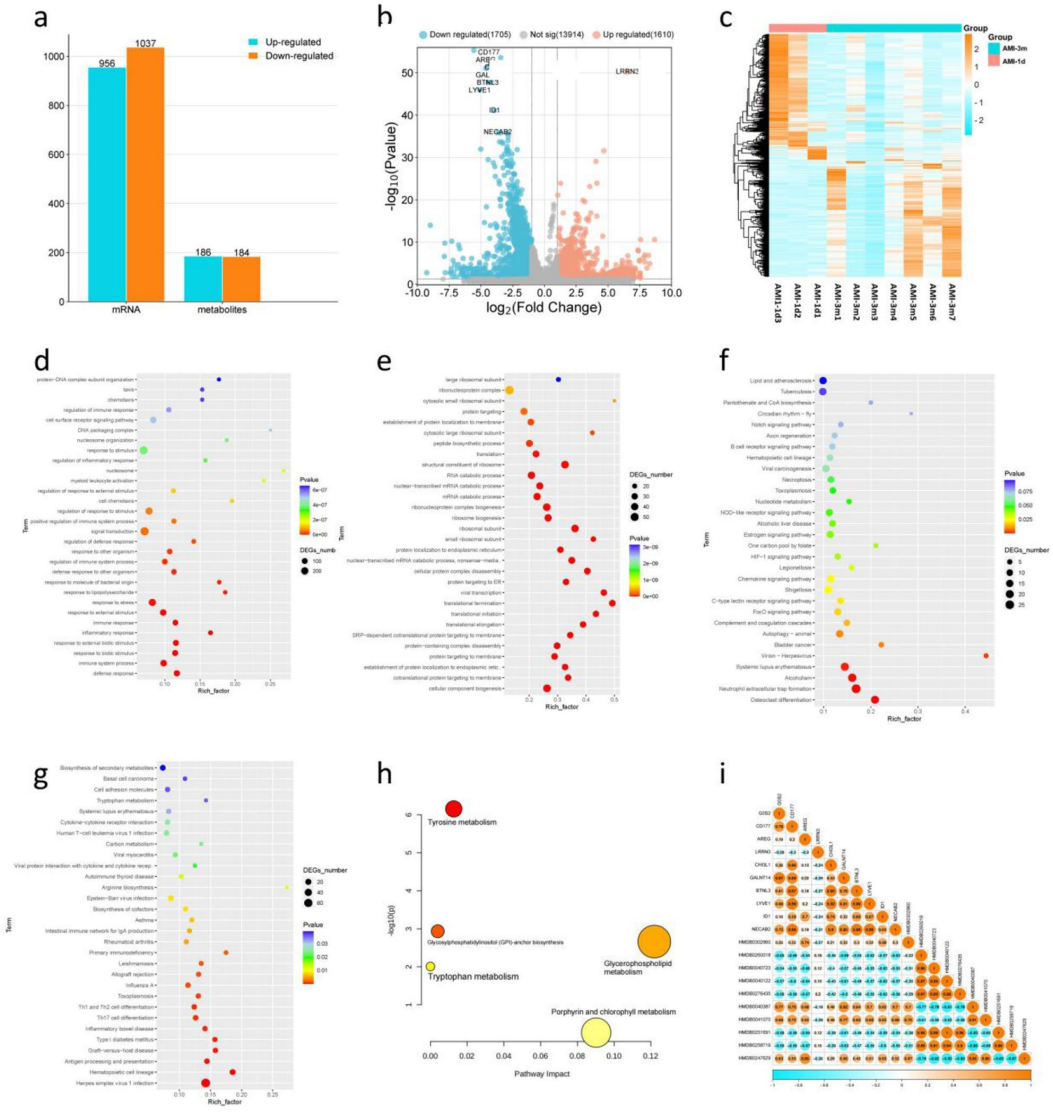
Transcriptional and metabolomic results between AMI-1d and AMI-3m: (**a**) Histogram of DEGs between AMI-1d and AMI-3m; (**b**) Volcano plot of DEGs between AMI-1d and AMI-3m; (**c**) Clustering heat map of DEGs betweenAMI-1d and AMI-3m; (**d**) Go enrichment analysis of up-regulated DEGs; (**e**) Go enrichment analysis of down-regulated DEGs; (**f**) KEGG enrichment analysis of up-regulated DEGs; (**g**) KEGG enrichment analysis of down-regulated DEGs; (**h**) KEGG enrichment analysis of DMs; (**i**) Correlation analysis between the top 10 DEGs and DMs.

Compared to AMI-1d, there were 956 upregulated genes and 1037 downregulated genes in AMI-3 m (Fig. 4A, Table S5). The top 10 genes ranked by P-value were G0S2, CD177, AREG, LRRN3, CHI3L1, GALNT14, BTNL3, LYVE1, ID1, and NECAB2 (Fig. 4B). The clustered heatmap analysis showed distinct differences in the gene expression between the two groups (Fig. 4C). To better understand the differences in biological functions and pathways between AMI-1d and AMI-3m, we performed enrichment analysis separately for the upregulated and downregulated DEGs. The results of GO enrichment showed that the downregulated genes in AMI-3m were enriched in biological processes such as defense response, immune system process, response to biotic stimulus, response to external biotic stimulus, immune response, inflammatory response, response to stress, response to molecule of bacterial origin, and response to lipopolysaccharide (Fig. 4D). The upregulated genes in AMI-3m were enriched in biological processes such as cellular component biogenesis, protein targeting to membrane, translational termination, translational elongation, translational initiation, SRP-dependent cotranslational protein targeting to membrane, protein-containing complex disassembly, cotranslational protein targeting to membrane, viral transcription, and establishment of protein localization to the endoplasmic reticulum (Fig. 4E). The KEGG enrichment analysis revealed that the downregulated genes in AMI-3 m were enriched in pathways such as autophagy—animal, complement and coagulation cascades, FoxO signaling pathway, C-type lectin receptor signaling pathway, nucleotide metabolism, NOD-like receptor signaling pathway, estrogen signaling pathway, and HIF-1 signaling pathway (Fig. 4F). The upregulated genes in AMI-3m were enriched in pathways such as herpes simplex virus 1 infection, hematopoietic cell lineage, graft-versus-host disease, antigen processing and presentation, type I diabetes mellitus, Th17 cell differentiation, inflammatory bowel disease, and Th1 and Th2 cell differentiation (Fig. 4G).

A total of 370 differential metabolites between AMI-3m and AMI-1d were identified (P-value < 0.05, VIP > 1), with 186 upregulated and 184 downregulated in AMI-3 m (Fig. 4A). The list of DMs can be found in Table S6. The top 10 metabolites ranked by P-value were HMDB0302960, HMDB0260018, HMDB0040723, HMDB0040122, HMDB0276435, HMDB0040387, HMDB0041070, HMDB0251691, HMDB0258719, and HMDB0247629. All DMs were imported into the MetaboAnalyst database for metabolic pathway analysis, resulting in 5 enriched metabolic pathways. These pathways include tryptophan metabolism, glycosylphosphatidylinositol (GPI)-anchor biosynthesis, glycerophospholipid metabolism, tryptophan metabolism, and porphyrin and chlorophyll metabolism (Fig. 4H). To analyze the relationship between the genome and metabolome, we selected the top 10 genes and metabolites based on their P-values and performed correlation analysis. The results of Pearson correlation analysis showed that there was a correlation coefficient greater than 0.8 between HMDB0040387 and GALNI14 (Fig. 4I).

## Discussion

Post-myocardial infarction ventricular remodeling and heart failure are focal points of concern for the prognosis of myocardial infarction patients. Following myocardial infarction, the process of cardiac repair and remodeling is accompanied by continuous changes in genotype and metabolic phenotype. Utilizing omics techniques to perform multi-time point analysis of peripheral blood after myocardial infarction may aid in our understanding of the biological process and future therapeutic approaches for the dynamic evolution of myocardial infarction.

Compared with AMI-1d, 876 genes and 119 metabolites were up-regulated, 1168 genes and 110 metabolites were down-regulated in AMI-7d. Compared with AMI-7d, AMI-3m had 245 up-regulated genes, 144 up-regulated metabolites, 443 down-regulated genes and 149 down-regulated metabolites. Compared with AMI-1d, AMI-3m had 956 up-regulated genes, 186 up-regulated metabolites, 1037 down-regulated genes and 184 down-regulated metabolites.

### Biological characteristics of AMI-1d

Compared to the AMI-7d, the acute phase of myocardial infarction (AMI-1d) showed significant upregulation in biological processes such as defense response and inflammatory response, as well as signaling pathways including Glycerophospholipid metabolism, Toll-like receptor signaling pathway, and IL-17 signaling pathway. This indicates that immune-inflammatory responses and glycerophospholipid metabolism signaling pathways are extensively activated in AMI-1d. The main metabolites in the Glycerophospholipid metabolism pathway are phosphatidylcholine (PC) and phosphatidylethanolamine (PE). It has been reported that plasma levels of PE, PC and other lipids are significantly higher in 21-month-old rabbits with myocardial infarction compared to normal rabbits^14^. Furthermore, serum lipidomics studies in clinical myocardial infarction and post-myocardial infarction heart failure have found that PE (12:1e_22:0) and PC (22:4_14:1) are early biomarkers that can effectively differentiate post-myocardial infarction heart failure. PE (12:1e_22:0) was inversely correlated with BNP and BUN while PC (22:4_14:1) was positively associated with both BNP and BUN^15^.

The abundant inflammation observed in AMI is primarily caused by the immune and inflammatory responses triggered by myocardial tissue necrosis^16^. Insufficient oxygen and nutrients lead to the necrosis of myocardial cells. The necrotic myocardial cells release a large amount of cell components and signaling molecules, such as intracellular enzymes, nuclear components, and inflammatory mediators^17^. Additionally, ischemia and reperfusion injury activate inflammatory responses and induce the clustering and activation of inflammatory cells, including neutrophils, monocytes, and macrophages. These substances can act as inflammatory signaling molecules, attracting and activating inflammatory cells. These inflammatory cells help remove necrotic tissue by engulfing necrotic cells and their fragments, but they also secrete inflammatory mediators involved in the regulation and repair processes of inflammation^18^. Neutrophil-associated pathways were found in patients with low CRP levels, indicating the presence of residual inflammatory risk beyond the traditional NLRP3 pathway^19^. In this study, within 24 h of myocardial infarction, signaling pathways such as Toll-like receptor signaling pathway and IL-17 signaling pathway were significantly upregulated. Animal experiments have shown the crucial role of IL-17 and Toll-like receptor in infarct injury, particularly in its adverse effects on ventricular remodeling^20,21^. Clinical trials have also indicated a correlation between high circulating levels of IL-17A and poor clinical prognosis^22^. The inflammation in the acute phase of myocardial infarction is beneficial, but if prolonged, it may result in severe cardiac damage. Therefore, anti-inflammatory therapy is an important target for preventing pathological changes associated with myocardial infarction.

Simultaneously, biological processes such as regulation of nucleic acid-templated transcription, regulation of transcription, DNA-templated, regulation of RNA biosynthetic process, and regulation of macromolecule biosynthetic process are downregulated, indicating a decrease in the physiological functions of cellular macromolecule biosynthesis, as well as anti-inflammatory responses, in AMI-1d. After myocardial infarction, the damage to cardiac cells due to ischemia and necrosis may lead to a decrease in protein synthesis capacity in cardiomyocytes. This could be attributed to factors such as reduced energy and nutrient supply to the cells, as well as inflammatory responses^23^. Additionally, cell injury and ischemia/reperfusion injury may disrupt the pathways involved in DNA and RNA synthesis within the cell nucleus, thereby affecting the synthesis rates of DNA and RNA^24^.

### Biological characteristics of AMI-7d

Compared to AMI-3m, pathways such as platelet activation, Complement and coagulation cascades, TGF-beta signaling pathway, and extracellular matrix (ECM) receptor interaction were upregulated in AMI-7d. This indicates that around 7 days after myocardial infarction, platelets are still in an activated state, and the cardiac fibrotic response begins to initiate^25,26^. Platelet activation, aggregation, and the formation of occlusive thrombi have been implicated in post-myocardial infarction microvascular obstruction and infarct expansion^27^. Our study found an activated platelet pathway during AMI-7d, particularly characterized by upregulation of GP1BA (glycoprotein Ib platelet subunit) and GP5 (glycoprotein V platelet). GP1BA is an important factor involved in platelet adhesion, while GP5 is one of the most abundant glycoproteins on the platelet membrane surface^28^. GPV is a new marker for platelet activation, and measuring GPV levels helps assess the effects of antithrombotic and antiplatelet drugs^29,30^.

Myocardial fibrosis is the process of extracellular ECM deposition between myocardial cells, leading to remodeling and stiffening of the heart tissue^31^. The extent of cardiac fibrosis predicts poor outcomes in most cardiac diseases. Abundant fibroblasts that can be activated after injury^32–36^ contribute to matrix remodeling by producing structural proteins and ECM proteins, playing a crucial role in scar maturation^37^.

TGF-β, a multifunctional cytokine, plays a significant role in the development of cardiac fibrosis, and the TGF-β/Smad3 pathway is a critical process for activating the phenotypes of matrix-producing cardiac fibroblasts and transforming fibroblasts into myofibroblasts^38,39^. Dead myocardial cells are replaced by collagen fibers secreted by fibroblasts, forming “reparative fibrosis”, while myocardium distal to the infarct area undergoes “reactive fibrosis” under the regulation of secreted factors, hormones, and mechanical stress, ultimately leading to myocardial stiffness and impaired diastolic function. This study confirms that pathways such as the TGF-beta signaling pathway and the interaction between ECM and cells are upregulated after 7 days of myocardial infarction, indicating the initiation of myocardial fibrosis response and the proliferation phase of cardiac repair.

### Biological characteristics of AMI-3m

AMI-3m showed upregulation of 245 DEGs, among which NOS3 and SHC2 were enriched in the VEGF signaling pathway. This suggests that the VEGF signaling pathway is significantly activated in the chronic phase after myocardial infarction. VEGF is a highly specific endothelial cell regulatory factor that promotes neovascularization and increases vascular permeability^40,41^. After myocardial infarction, myocardial cells, macrophages, fibroblasts, and other cells produce a large amount of VEGF to regulate endothelial cell function, which helps in the rescue of myocardial^42,43^. Previous studies have shown that circulating NOS3 modulates left ventricular remodeling following reperfused myocardial infarction^44,45^. VEGF stimulates SHC phosphorylation and ultimately control vasopermeability and angiogenesis.

Angiogenesis is the process of forming new blood vessels, which enhances oxygen and nutrient supply by increasing new vascular networks. This process promotes the repair of necrotic areas and regeneration of myocardial tissue^46^. In mice, 2–4 days after myocardial infarction, capillary networks in the border zone begin to expand, with numerous branches and blood vessels entering the center of the infarction. By day 7, most of the endothelial cells in the border zone stop proliferating, and the newly formed capillaries enlarge with the support of smooth muscle cells^47^. In pigs, the ischemic area showed a coordinated upregulation of angiogenesis signaling processes at early reperfusion (day 4 and 7)^48^, while in the peri-infarct border zone, VEGF signaling indicating active angiogenesis upregulated at 1 month after AMI.

Anyway, humans usually do not develop pathological remodeling in days or weeks^49^, the growth of blood vessels is most pronounced at than in mice or pigs^50^. This study confirm that around 3 months after myocardial infarction, there is an upregulation of NOS3 and SHC2. This indicates that the proliferation, migration, and vascular formation of endothelial cells mediated by the VEGF pathway are important biological characteristics of the chronic phase of myocardial infarction. Some clinical trials have utilized adenoviral vectors^51^, non-viral plasmids^52^, or recombinant proteins^53^ to deliver VEGF for the treatment of cardiovascular diseases. Although some of these trials have shown moderate improvements in cardiac function, the clinical translation has been limited due to the limitations of VEGF, such as its short plasma half-life (about 30 min), susceptibility to proteolytic degradation, immunogenicity, and high production costs^54^. VEGF, as a molecular probe and nanomedicine, holds promise for providing new therapeutic approaches for acute myocardial infarction.

## Conclusion

In order to characterize the molecular features of dynamic evolution in acute myocardial infarction, we collected blood samples at approximately 1 day, 7 days, and 3 months after myocardial infarction. Transcriptomic and metabolomic sequencing were performed on these samples. Through bioinformatic analysis, we discovered key pathological processes at different stages following myocardial infarction.

The biological characteristics of the acute phase of MI (AMI-1d) are primarily acute inflammatory response, elevated glycerophospholipid metabolism and decreased protein synthesis capacity. The biological characteristics of the subacute phase of MI (AMI-7d) are mainly initiation of myocardial fibrosis response and platelet overactivation. The biological characteristics of old myocardial infarction (AMI-3m) mainly involve vascular regeneration response.

However, our study also has some limitations. We selected three time points after myocardial infarction, and due to the limited time points, the pathological changes in the dynamic evolution of myocardial infarction may not be precisely captured. In addition, we could not ensure consistent sample sizes at different time points. To facilitate more targeted intervention measures, further in vivo and in vitro experiments are needed to gain a deeper understanding of post-AMI.

## Materials and methods

### Patient recruitment

From June 2022 to May 2023, a total of 95 participants undergoing AMI were recruited from the wards of Guang’anmen Hospital and Langfang People’s Hospital. According to the guidelines^55,56^, AMI participants confirmed by coronary angiography were included, with ages ranging from 30 to 70 years, and willing to sign informed consent forms. Exclusion criteria included poorly controlled hypertension, severe cardiopulmonary insufficiency, severe cardiac arrhythmias, as well as participants with severe primary diseases such as liver, kidney, hematopoietic system disorders. Participants with neurological or psychiatric disorders who were unable or unwilling to cooperate, as well as pregnant or lactating women, were also excluded. This study was approved by ethics committee of Guang’anmen Hospital (2022-003-KY). It was conducted in accordance with ethical standards established by the National Research Committee and the 1964 Helsinki Declaration and its subsequent revisions or similar ethical standards. Informed consent was obtained from all volunteers before any research procedures were conducted.

### RNA-seq approach

#### Sample collections

15 participants with myocardial infarction were enrolled from the Guang’anmen Hospital. Among them, there were 3 participants were AMI-1d group (within 1 day after myocardial infarction), 5 participants were AMI-7d group (within 7 days ± 1 day after myocardial infarction), and 7 participants are AMI-3m group (within 12 weeks ± 1 week after myocardial infarction). In the early morning, the researchers collected peripheral blood samples from the participants in vacuum tubes containing EDTA anticoagulant. Total RNA was extracted from the tissue using TRIzol® Reagent according the manufacturer’s instructions (Invitrogen) and genomic DNA was removed using DNase I (TaKara). Then RNA quality was determined using 2100 Bioanalyser (Agilent) and quantified using the ND-2000 (NanoDrop Technologies). High-quality RNA sample (OD260/280 = 1.8–2.2, OD260/230 ≥ 2.0, RIN ≥ 6.5, 28S:18S ≥ 1.0, > 10 μg) is used to construct sequencing library.

#### Library preparation, and Illumina Hiseq sequencing

RNA-seq transcriptome libraries were prepared following TruSeqTM RNA sample preparation Kit from Illumina (San Diego, CA), using 1 μg of total RNA. Shortly, messenger RNA was isolated with polyA selection by oligo (dT) beads and fragmented using fragmentation buffer. cDNA synthesis, end repair, A-base addition and ligation of the Illumina-indexed adaptors were performed according to Illumina’s protocol. Libraries were then size selected for cDNA target fragments of 200–300 bp on 2% Low Range Ultra Agarose followed by PCR amplified using Phusion DNA polymerase (NEB) for 15 PCR cycles. After quantified by TBS380, Paired-end libraries were sequenced by Illumina NovaSeq 6000 sequencing (150 bp*2, Shanghai BIOZERON Co., Ltd).

#### Differential gene expression analysis and functional enrichment

The raw paired end reads were trimmed and quality controlled by Trimmomatic with parameters (SLIDINGWINDOW:4:15 MINLEN:75). Then clean reads were separately aligned to reference genome with orientation mode using hisat2 software. This software was used to map with default parameters. The quality assessment of these data were taken by qualimap_v2.2.1. Use htseq (https://htseq.readthedocs.io/en/release_0.11.1/)to count each gene reads.

To identify DEGs (differential expression genes) between the two different samples, the expression level for each gene was calculated using the fragments per kilobase of exon per million mapped reads (FRKM) method.R statistical package edgeR (Empirical analysis of Digital Gene Expression in R, http://www.bioconductor.org/packages/release/bioc/html/edgeR.html/) was used for differential expression analysis. The DEGs between two samples were selected using the following criteria: the logarithmic of fold change was greater than 2 and the false discovery rate (FDR) should be less than 0.05. To understand the functions of the differentially expressed genes (DEGs), GO functional enrichment and KEGG pathway analysis were carried out by Goatools (https://github.com/tanghaibao/Goatools) and KOBAS (http://kobas.cbi.pku.edu.cn/home.do). DEGs were significantly enriched in GO terms and metabolic pathways when their Bonferroni-corrected P-value was less than 0.05.

#### Metabolomics methods

95 participants with myocardial infarction came from Guang’anmen Hospital and Langfang People’s Hospital. Among them, 33 were AMI-1d group, 33 were AMI-7d group, and 29 were AMI-3m group. Patient blood samples were collected after fasting for 12 h and in vacuum tubes containing EDTA anticoagulants. Metabolomics methods were used according to previous researches^57,58^. Mixed the serum (0.1 mL) with 0.9 mL 80% methanol containing 0.1% formic acid (FA). After being vortexed for 30 s and treated with ultrasonic for 20 min, all samples were frozen for 1 h at − 20 °C for protein precipitation. Then the samples were centrifuged with 12,000×*g* at 4 °C for 10 min, each supernatant (800 μL) was obtained and transferred to a sample vial. Quality control (QC) samples were prepared by pooling aliquot of all serum samples for serum metabolomics analysis. Blank sample (80% methanol containing 0.1% formic acid) and QC sample were repeated every ten samples during data acquisition. The sample vials were stored at − 20 °C until detection.

The UPLC-QTOF MS analysis were performed using a UPLC system (ACQUITY UPLC I-Class, Waters) coupled to an electrospray ionization quadruple time-of-flight mass spectrometer (ESI-QTOF MS) (SYNAPT G2-Si HDMS, Waters). A Waters ACQUITY BEH C18 column (1.7 μm; 100 mm × 2.1 mm) was used for LC separation and the column temperature was kept at 40 °C. The flow rate was 0.4 mL/min and the sample injection volume was 10 μL. The mobile phase A was 0.1% FA in water, and mobile phase B was 0.1% FA in ACN. The linear gradient was set as follows: Initial to 1 min: 10% B, 0–3 min 10% B to 80% B, 3–8 min: 95% B to 95% B, 8.1–10 min: 10% B.

High-accuracy MS data were recorded by MassLynx 4.1 software. Capillary voltage was 2.5 kV for positive mode and 2 kV for negative mode, whereas cone voltage was 30 V for both modes. The source temperature was set at 120 °C with a cone gas flow of 50 L/h, and the desolvation temperature was set at 500 °C with a desolvation gas flow of 800 L/h. Leucine-enkephalin (LE) was used as the lock mass generating a reference ion at m/z 556.2771 in positive mode and m/z 554.2615 in negative mode, which was introduced by a lockspray at 10 μL/min for data calibration. The MSE data were acquired in continuum mode using ramp collision energy in two scan functions. For low energy mode, scan range 50–1200 Da, scan time 0.2 s, and collision energy 6 V were set. For high energy, scan range 50–1200 Da, scan time 0.2 s, and a collision energy ramp 15–45 V were set.

Raw data were imported to commercial software Progenesis QI (Version 2.4, Waters) for data processing, which included peak picking, peak alignment and acquiring compound-associated information such as m/z, retention time and intensity. Next, data filtering was performed to delete low-quality data. Ions with a relative standard deviation (RSD) of more than 30% in quality control (QC) samples were filtered. These filtered ions fluctuated too much among samples and were not included for further analysis. OPLS-DA (Partial least squares Discriminant Analysis) was performed and VIP (variable importance in projection) was calculated by MetaboAnalyst 5.0 (https://www.metaboanalyst.ca/)^59^ and R project. Metabolites with P < 0.05 and VIP > 1 were selected as differentially expressed metabolites (DMs). R project was also applied in further data processing, statistical analysis. Pathway analysis was performed by MetaboAnalyst 5.0.

### Supplementary Information

Supplementary Tables.

## Data Availability

We have submitted the raw RNA-seq data to GEO (https://www.ncbi.nlm.nih.gov/geo/subs/) under the accession number GSE249812. Besides, we have uploaded PLC/LC–MS data to the MetaboLights (https://www.ebi.ac.uk/metabolights/editor/study/MTBLS9088/descriptors) with the number of MTBLS9088.

## References

[1] WHO (2016). World Health Statistics 2016: Monitoring Health for the SDGs Sustainable Development Goals.

[2] Vaduganathan, M., Mensah, G. A., Turco, J. V., Fuster, V. & Roth, G. A. The global burden of cardiovascular diseases and risk: a compass for future health. Vol. 80, 2361–2371 (American College of Cardiology Foundation, 2022). 10.1016/j.jacc.2022.11.005 36368511

[3] Stamatis P, Turkiewicz A, Englund M, Turesson C, Mohammad AJ (2022). Epidemiology of biopsy-confirmed giant cell arteritis in southern Sweden—An update on incidence and first prevalence estimate. Rheumatology.

[4] Collet JP (2021). 2020 ESC Guidelines for the management of acute coronary syndromes in patients presenting without persistent ST-segment elevation. Eur. Heart J..

[5] Levine GN (2016). 2015 ACC/AHA/SCAI focused update on primary percutaneous coronary intervention for patients with ST-elevation myocardial infarction: An update of the 2011 ACCF/AHA/SCAI guideline for percutaneous coronary intervention and the 2013 ACCF/AHA guideline for the management of ST-elevation myocardial infarction: A report of the American College of Cardiology/American Heart Association Task Force on Clinical Practice Guidelines and the Society for Cardiovascular Angiography and Interventions. Circulation.

[6] Gerber Y (2016). Mortality associated with heart failure after myocardial infarction: A contemporary community perspective. Circ. Heart Fail..

[7] Ibanez B (2018). 2017 ESC Guidelines for the management of acute myocardial infarction in patients presenting with ST-segment elevation: The Task Force for the management of acute myocardial infarction in patients presenting with ST-segment elevation of the European Society of Cardiology (ESC). Eur. Heart J..

[8] Talman V, Ruskoaho H (2016). Cardiac fibrosis in myocardial infarction—From repair and remodeling to regeneration. Cell Tissue Res..

[9] Vafadarnejad E (2020). Dynamics of cardiac neutrophil diversity in murine myocardial infarction. Circ. Res..

[10] Nam M, Jung Y, Hwang G-S (2017). A metabolomics-driven approach reveals metabolic responses and mechanisms in the rat heart following myocardial infarction. Int. J. Cardiol..

[11] Silbiger VN (2011). Time course proteomic profiling of human myocardial infarction plasma samples: An approach to new biomarker discovery. Clin. Chim. Acta.

[12] Ward-Caviness CK (2018). Analysis of repeated leukocyte DNA methylation assessments reveals persistent epigenetic alterations after an incident myocardial infarction. Clin. Epigenet..

[13] Zhang L (2021). Metabolomics reveal dynamic changes in eicosanoid profile in patients with ST-elevation myocardial infarction after percutaneous coronary intervention. Clin. Exp. Pharmacol. Physiol..

[14] Fiorelli S (2021). Lipidomics analysis of monocytes from patients with acute myocardial infarction reveals lactosylceramide as a new player in monocyte migration. FASEB J..

[15] Rong J, He T, Zhang J, Bai Z, Shi B (2023). Serum lipidomics reveals phosphatidylethanolamine and phosphatidylcholine disorders in patients with myocardial infarction and post-myocardial infarction-heart failure. Lipids Health Dis..

[16] Li T (2023). Cardiac repair after myocardial infarction: A two-sided role of inflammation-mediated. Front. Cardiovasc. Med..

[17] Fonseca FA, Izar MC (2022). Role of inflammation in cardiac remodeling after acute myocardial infarction. Front. Physiol..

[18] Peet C, Ivetic A, Bromage DI, Shah AM (2020). Cardiac monocytes and macrophages after myocardial infarction. Cardiovasc. Res..

[19] Nurmohamed NS (2022). Targeted proteomics improves cardiovascular risk prediction in secondary prevention. Eur. Heart J..

[20] Yan X (2012). Deleterious effect of the IL-23/IL-17A axis and γδT cells on left ventricular remodeling after myocardial infarction. J. Am. Heart Assoc..

[21] Zhou S-F (2014). IL-17A promotes ventricular remodeling after myocardial infarction. J. Mol. Med..

[22] Zhu F (2011). IL-17 induces apoptosis of vascular endothelial cells—A potential mechanism for human acute coronary syndrome. Clin. Immunol..

[23] Wang W (2016). Defective branched chain amino acid catabolism contributes to cardiac dysfunction and remodeling following myocardial infarction. Am. J. Physiol. Heart Circ. Physiol..

[24] Rout A, Tantry US, Novakovic M, Sukhi A, Gurbel PA (2020). Targeted pharmacotherapy for ischemia reperfusion injury in acute myocardial infarction. Expert Opin. Pharmacother..

[25] Ruggeri ZM (2002). Platelets in atherothrombosis. Nat. Med..

[26] Gurbel PA, Jeong Y-H, Navarese EP, Tantry US (2016). Platelet-mediated thrombosis: From bench to bedside. Circ. Res..

[27] Qi Z (2021). PCSK9 (proprotein convertase subtilisin/kexin 9) enhances platelet activation, thrombosis, and myocardial infarct expansion by binding to platelet CD36. Circulation.

[28] Dib F (2022). Biological, clinical features and modelling of heterozygous variants of glycoprotein Ib platelet subunit alpha (GP1BA) and glycoprotein Ib platelet subunit beta (GP1BB) genes responsible for constitutional thrombocytopenia. Br. J. Haematol..

[29] Beck S (2023). Platelet glycoprotein V spatio-temporally controls fibrin formation. Nat. Cardiovasc. Res..

[30] Ravanat C (2000). GPV is a marker of in vivo platelet activation—Study in a rat thrombosis model. Thromb. Haemost..

[31] Frangogiannis NG (2021). Cardiac fibrosis. Cardiovasc. Res..

[32] Zeisberg EM (2007). Endothelial-to-mesenchymal transition contributes to cardiac fibrosis. Nat. Med..

[33] Aisagbonhi O (2011). Experimental myocardial infarction triggers canonical Wnt signaling and endothelial-to-mesenchymal transition. Dis. Models Mech..

[34] Haudek SB (2006). Bone marrow-derived fibroblast precursors mediate ischemic cardiomyopathy in mice. Proc. Natl. Acad. Sci. U.S.A..

[35] Möllmann H (2006). Bone marrow-derived cells contribute to infarct remodelling. Cardiovasc. Res..

[36] Haider N (2019). Transition of macrophages to fibroblast-like cells in healing myocardial infarction. J. Am. Coll. Cardiol..

[37] Venugopal H, Hanna A, Humeres C, Frangogiannis NG (2022). Properties and functions of fibroblasts and myofibroblasts in myocardial infarction. Cells.

[38] Frangogiannis N (2020). Transforming growth factor-β in tissue fibrosis. J. Exp. Med..

[39] Dobaczewski M (2010). Smad3 signaling critically regulates fibroblast phenotype and function in healing myocardial infarction. Circ. Res..

[40] Nishibe T (2010). Expression and localization of vascular endothelial growth factor in normal abdominal aorta and abdominal aortic aneurysm. Int. Angiol..

[41] Holmes K, Roberts OL, Thomas AM, Cross MJ (2007). Vascular endothelial growth factor receptor-2: Structure, function, intracellular signalling and therapeutic inhibition. Cell. Signal..

[42] Kobayashi K (2017). Dynamics of angiogenesis in ischemic areas of the infarcted heart. Sci. Rep..

[43] Ferraro B (2019). Pro-angiogenic macrophage phenotype to promote myocardial repair. J. Am. Coll. Cardiol..

[44] Merx MW (2014). Depletion of circulating blood NOS3 increases severity of myocardial infarction and left ventricular dysfunction. Basic Res. Cardiol..

[45] Gorressen S (2015). Circulating NOS3 modulates left ventricular remodeling following reperfused myocardial infarction. PLoS ONE.

[46] Martín-Bórnez M (2023). New insights into the reparative angiogenesis after myocardial infarction. Int. J. Mol. Sci..

[47] Dubé KN (2017). Recapitulation of developmental mechanisms to revascularize the ischemic heart. JCI Insight.

[48] Binek A (2017). Proteomic footprint of myocardial ischemia/reperfusion injury: Longitudinal study of the at-risk and remote regions in the pig model. Sci. Rep..

[49] Akpalu D (2017). Matrix signaling subsequent to a myocardial infarction: A proteomic profile of tissue factor microparticles. JACC Basic Transl. Sci..

[50] Mallory GK, White PD, Salcedo-Salgar J (1939). The speed of healing of myocardial infarction: A study of the pathologic anatomy in seventy-two cases. Am. Heart J..

[51] Povsic TJ (2021). Epicardial delivery of XC001 gene therapy for refractory angina coronary treatment (The EXACT Trial): Rationale, design, and clinical considerations. Am. Heart J..

[52] Anttila V (2020). Synthetic mRNA encoding VEGF-A in patients undergoing coronary artery bypass grafting: Design of a phase 2a clinical trial. Mol. Ther. Methods Clin. Dev..

[53] Henry TD (2003). The VIVA trial: Vascular endothelial growth factor in ischemia for vascular angiogenesis. Circulation.

[54] Li B, Li Y, Chen S, Wang Y, Zheng Y (2023). VEGF mimetic peptide-conjugated nanoparticles for magnetic resonance imaging and therapy of myocardial infarction. J. Control. Release.

[55] Collet J-P (2021). 2020 ESC Guidelines for the management of acute coronary syndromes in patients presenting without persistent ST-segment elevation: The Task Force for the management of acute coronary syndromes in patients presenting without persistent ST-segment elevation of the European Society of Cardiology (ESC). Eur. Heart J..

[56] Levine GN (2016). 2015 ACC/AHA/SCAI focused update on primary percutaneous coronary intervention for patients with ST-elevation myocardial infarction: An update of the 2011 ACCF/AHA/SCAI guideline for percutaneous coronary intervention and the 2013 ACCF/AHA guideline for the management of ST-elevation myocardial infarction. J. Am. Coll. Cardiol..

[57] Wang L, Naser FJ, Spalding JL, Patti GJ (2019). A protocol to compare methods for untargeted metabolomics. Methods Mol. Biol..

[58] Dunn WB (2011). Procedures for large-scale metabolic profiling of serum and plasma using gas chromatography and liquid chromatography coupled to mass spectrometry. Nat. Protoc..

[59] Pang Z (2022). Using MetaboAnalyst 5.0 for LC-HRMS spectra processing, multi-omics integration and covariate adjustment of global metabolomics data. Nat. Protoc..

